# Integrated LC-MS/MS and Transcriptome Sequencing Analysis Reveals the Mechanism of Color Formation During Prickly Ash Fruit Ripening

**DOI:** 10.3389/fnut.2022.847823

**Published:** 2022-03-16

**Authors:** Xitong Fei, Yuan Wei, Yichen Qi, Yingli Luo, Haichao Hu, Anzhi Wei

**Affiliations:** ^1^College of Forestry, Northwest Agriculture and Forestry University, Xianyang, China; ^2^Research Centre for Engineering and Technology of Zanthoxylum State Forestry Administration, Xianyang, China; ^3^Frontiers Science Center for Flexible Electronics, Xi'an Institute of Flexible Electronics (IFE) and Xi'an Institute of Biomedical Materials & Engineering, Northwestern Polytechnical University, Xi'an, China

**Keywords:** fruit color, WGCNA, flavonoid synthesis pathway, anthocyanins, ANS, UFGT

## Abstract

Prickly ash peel is one of the eight major condiments in China and is widely used in cooking because of its unique fragrance and numbing taste. The color of prickly ash fruit is the most intuitive quality that affects consumer choice. However, the main components and key biosynthetic genes responsible for prickly ash fruit color have not yet been determined. To better understand the biosynthetic mechanisms and accumulation of prickly ash fruit color components, we performed an integrated transcriptomic and metabolomic analysis of red and green prickly ash fruit at different growth periods. The transcriptome analysis identified 17,269 differentially expressed genes (DEGs) between fruit of red and green prickly ash: 7,236 upregulated in green fruit and 10,033 downregulated. Liquid chromatography tandem mass spectrometry (LC-MS/MS) identified 214 flavonoids of 10 types. Flavonoids and flavonols are the main flavonoids in prickly ash, and the total flavonoid content of red prickly ash is higher than that of green prickly ash. Comprehensive analysis showed that the main colored metabolites that differed between green and red prickly ash were cyanidin-3-O-galactoside and cyanidin-3-O-glucoside, and differences in the contents of these metabolites were due mainly to differences in the expression of *ANS* and *UFGT*. Our results provide insight into the mechanisms underlying color differences in red and green prickly ash and will be useful for improving the quality of prickly ash fruit.

## Introduction

As a member of the Rutaceae family, prickly ash is one of the most economically valuable tree species in China. It originated in China and is distributed mainly in temperate and subtropical regions (1). The peel of prickly ash is one of the eight major condiments in China; it can stimulate the mouth and taste buds to produce a numbing sensation, and its rich aroma gives dishes a unique flavor (2). The young leaves and shoots of prickly ash are used as early spring vegetables and can also be made into canned food for long term preservation. In addition, prickly ash peels are a traditional Chinese medicine with pain-relieving, bacteriostatic, anti-inflammatory, antitussive, and expectorant properties (3). Based on the color of the fruit at commodity maturity, prickly ash can be divided into green prickly ash and red prickly ash. Green prickly ash and red prickly ash not only differ in color but also have significant differences in flavor, and consumers select different prickly ash according to their cooking preferences. Therefore, studying the biosynthetic mechanism(s) responsible for prickly ash fruit color can promote the continued development of the prickly ash industry. At present, the key regulatory genes that determine the difference in prickly ash fruit color remain unclear.

Anthocyanins are important flavonoid secondary metabolites found mainly in the form of glycosides in the vacuoles of flowers, fruits, seeds, leaves, and other organs of seed plants (4). Anthocyanins give plant tissues rich colors, which attract insects and animals to aid pollination and reproduction (5). They are highly effective antioxidants and can protect plants from biotic and abiotic stresses such as pathogen invasion, drought, strong light, low temperature, phosphorus deficiency and others (6, 7). They can also effectively scavenge free radicals and reactive oxygen species during plant stress (8), thereby improving plant stress tolerance (9). Anthocyanins can improve the postharvest preservation of fruits and vegetables. For instance, tomato fruit that are rich in anthocyanins have a longer quality guarantee period (10, 11). In addition, anthocyanins have a wide range of applications in clinical medicine, and have been shown to effectively minimize the risk of cancer and diabetes (12). For example, delphisine derivatives are the main anthocyanins in solanaceous vegetables and can reduce vascular inflammation and prevent thrombosis (13). Flavonoids are important antimicrobial agents in plants, and their biosynthesis can prevent the spread of pathogens (14). Because some flavonoids have antioxidant, anti-inflammatory, anti-allergic, anti-cancer, and antiviral properties, they are highly valued by the pharmaceutical and health care industry (15). For example, the use of flavonoids as synergists can improve the antibacterial efficiency of antibiotics (16).

Anthocyanin synthesis occurs through the flavonoid pathway. Phenylalanine ammonia-lyase (PAL) is the first enzyme in the pathway and catalyzes the synthesis of cinnamic acid from phenylalanine (17). Cinnamic acid is then used to synthesize anthocyanins through the activities of cinnamic acid hydroxylase (C4H), 4-coumarate-CoA ligase (4CL), chalcone synthase (CHS), chalcone isomerase (CHI), flavanone 3-hydroxylase (F3H), flavonoid 3′-monooxygenase (F3′H), flavonoid 3′,5'-hydroxylase (F3′5′H), bifunctional dihydroflavonol 4-reductase (DFR), anthocyanidin synthase (ANS), and UDP-glucose flavonoid 3-O-glucosyl transferase (UFGT) (18–20). Anthocyanin content is directly related to regulatory factors and the expression of flavonoid biosynthesis genes, and the study of this pathway is therefore useful for understanding the mechanisms of anthocyanin synthesis in prickly ash fruit.

In this study, liquid chromatography tandem mass spectrometry (LC-MS/MS) and transcriptome sequencing were used to analyze the color formation mechanism in both kinds of prickly ash fruit during different growth periods. Weighted gene co-expression network analysis (WGCNA) was used to identify gene modules strongly correlated with flavonoid content, and gene clusters related to flavonoid synthesis were screened. Analyzing differentially expressed genes and differentially abundant metabolites from the flavonoid synthesis pathway helps to explain the potential mechanism underlying the color difference in red and green prickly ash fruit. The results of this study provide reference data for the development of functional foods and medicines from prickly ash.

## Materials and Methods

### Prickly Ash Fruit Materials

Red prickly ash (*Zanthoxylum bungeanum*) and green prickly ash (*Zanthoxylum armatum*) were collected at different growth periods from the Chinese prickly ash experimental station (Northwest A&F University, Fengxian, China, N33°59′6.55′′ E106°39′29.38′′). The samples were taken from 7-year-old prickly ash trees grown under the same climatic conditions and management regime. The red prickly ash fruit were collected at three different growth periods: R1 (expansion), R2 (color conversion), and R3 (maturity) at 30, 60, and 90 days after flowering, respectively. Green fruit were collected at the same three stages: G1, G2, and G3, which also represent 30, 60, and 90 days after flowering. Nine trees with consistent growth were selected at each developmental stage, and every three fruits were mixed together as a biological replicate. There were three biological replicates in each developmental stage, and more than 100 fruits were collected from each tree. After collection, samples were quickly frozen in liquid nitrogen and stored at −80°C for subsequent use. The voucher specimen is kept in the Fengxian *Zanthoxylum bungeanum* Experimental Station of Northwest Agriculture and Forestry University.

### Extraction of Metabolites From Fruit

Red and green prickly ash fruit were vacuum freeze-dried (Scientz-100F), and powdered samples (100 mg) were extracted in 70% methanol. The samples were vortexed every 30 min for 30 s. After six rounds of vortexing, the samples were placed overnight in a 4°C refrigerator and then centrifuged (12,000 rpm, 10 min). The supernatant was passed through a microporous membrane (0.22 μm pore size) into an injection bottle for subsequent metabolome analysis (21).

### UPLC-MS/MS Analysis

The flavonoids in two kinds of prickly ash fruit were analyzed by UPLC-MS/MS (ultra-performance liquid chromatography, Shimadzu, Kyoto, Japan; mass spectrometry, 4,500 QTRAP, Thermo Scientific) using an Agilent SB-C18 column (1.8 μm, 2.1 mm × 100 mm). The mobile phase was made up of phase A (0.1% formic acid in ultrapure water) and phase B (0.1% formic acid in acetonitrile). The column temperature was 40°C, and the flow rate was set to 0.35 ml/min. The sample injection volume was 4 μl. The elution gradient began with 5% phase B at 0.00 min. The proportion of phase B increased to 95% at 9.00 min and was maintained for 1 min; it was then decreased to 5% from 10.00–11.10 min and remained at 5% until 14 min. Analyst 1.6.3 software (AB Sciex) was used to control the working of positive and negative ions. The source temperature was 550°C, and the ion spray voltage (IS) generated −4,500 V negative ions and 5,500 V positive ions. The m/z range used in the LC-MS/MS analysis was 50–1250 Da (22). A total of 18 samples were analyzed by LC-MS/MS: three biological replicates of six sample types.

### Analysis of Differentially Abundant Metabolites

The HCA (hierarchical clustering analysis) results for samples and metabolites were presented as heatmaps with dendrograms, whereas Pearson correlation coefficients (PCCs) between the samples were calculated using the cor function in R and presented only as heatmaps. Both HCA and PCC were carried out with the R package pheatmap. For HCA, normalized signal intensities of metabolites (unit variance scaling) were visualized as a color spectrum. Principal component analysis (PCA) was used to analyze the metabolomics data and identify differentially abundant metabolites between the different varieties. Integrating fold change and variable importance in projection (VIP) values, thresholds of fold change ≥ 2 or fold change ≤ 0.5 and VIP ≥ 1 were used to identify significantly different metabolites. To avoid overfitting, a permutation test (200 permutations) was performed. KEGG pathway enrichment analysis was conducted on the differentially abundant metabolites to identify the main metabolic processes with which they were associated (23).

### DEG Expression Pattern Clustering

STEM software was used to cluster the DEGs according to their expression patterns in the transcriptome data (24). Gene expression level was normalized as log_2_ (FPKM), the K-means clustering method was used, *p*-values were corrected based on an FDR of 0.05, and the minimum number of clustered genes was set to 20.

### RNA Extraction and Sequencing

The MiniBEST Plant RNA Extraction Kit (TaKaRa, China) was used to extract total RNA from prickly ash fruit of different growth periods. The concentration and quality of total RNA samples were measured and calculated with a NanoDrop 2000 spectrophotometer (Thermo Scientific, Pittsburgh, PA, USA), and the optimal total RNA from each sample was used to construct a cDNA library for transcriptome sequencing. Eighteen libraries were constructed and sequenced on the NovaSeq 6000 platform (Illumina, San Diego, USA).

### Functional Annotation and Identification of Differentially Expressed Genes

Unigenes were annotated based on the results of BLAST searches against the COG, NR, Pfam, GO, KOG, KEGG and Swiss-Prot databases, and their predicted amino acid sequences were used in an HMMER search of the Pfam database (25). Expression of unigenes was computed as fragments per kilobase per million mapped reads (FPKM), and differentially expressed unigenes were identified based on a threshold of fold change ≥ 2.0 and *p* value < 0.05 in the DESeq R package (26).

### Co-expression Analysis (WGCNA)

The WGCNA R package (v1.6.6) provides a series of functions for constructing a weighted gene co-expression network and partitioning related modules (27). Differentially expressed genes (DEGs) and differentially abundant flavonoids (DAFs) from two kinds of prickly ash fruit at different growth periods were analyzed to identify genes related to flavonoid synthesis. The pickSoftThreshold function was used to select the best soft thresholding power for network building. The blockwiseModules function and default parameters were used to structure a scale-free co-expression network, and the network was then visualized using Cytoscape (v3.5.1) (28). KEGG enrichment analysis was also performed (29).

### Canonical Correlation Analysis (CCA)

As a common analysis method, canonical correlation analysis (CCA) is used to investigate the relationships between response variables and explanatory variables and can perform dimensionality reduction analysis on explanatory variables to identify those that play a major role. We used Canoco software to perform correlation analysis on the flavonoid content and gene expression levels of the two kinds of prickly ash fruit from different growth periods and to identify key genes involved in flavonoid synthesis.

## Results

### The Accumulation of Flavonoids in Red and Green Prickly Ash During Fruit Development

**Figure 1:**
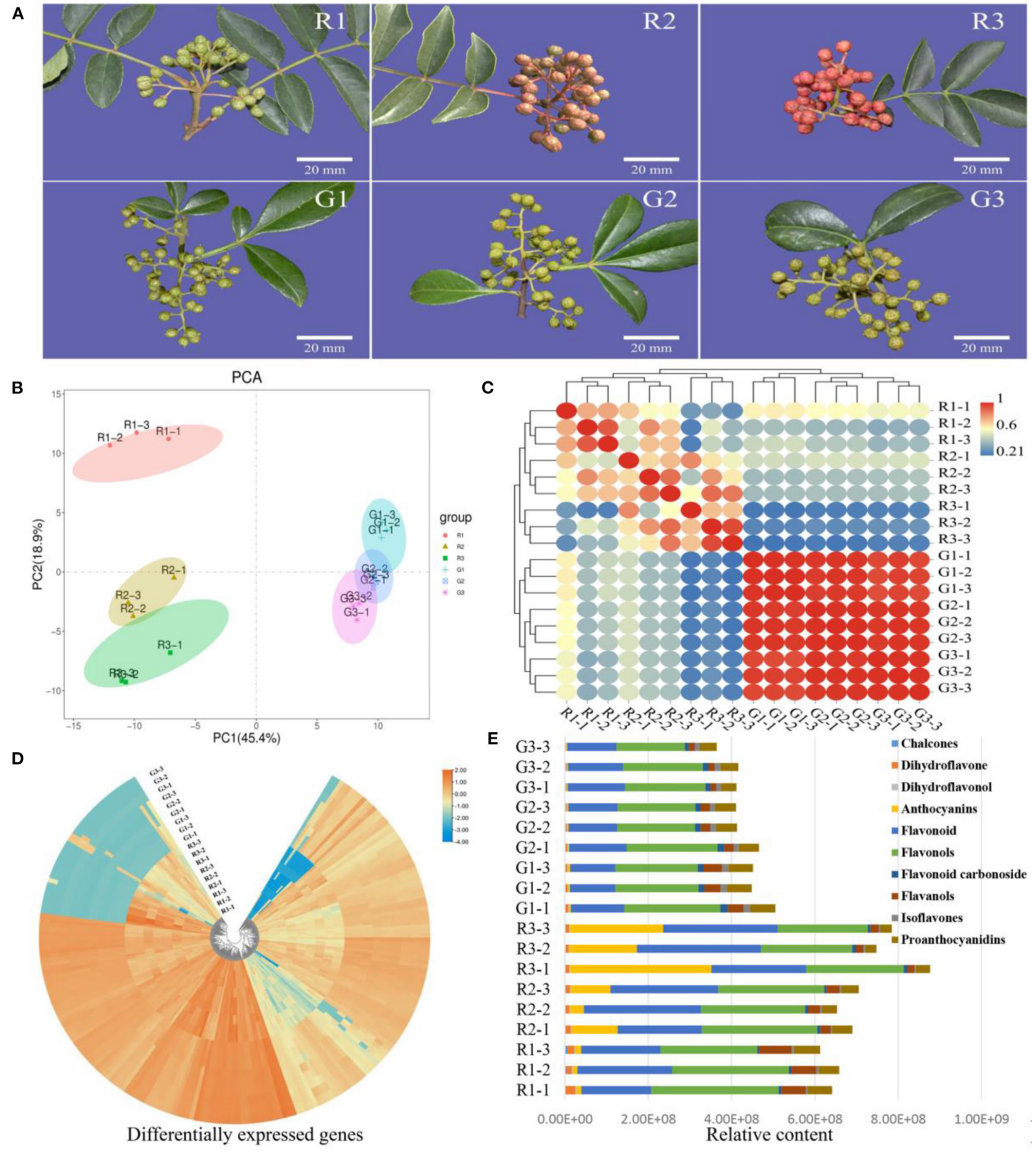
Metabolite analysis of red and green prickly ash fruit at different growth periods. **(A)** Red and green prickly ash fruit at different growth periods. The scale bar is 20 mm. **(B)** PCA analysis of flavonoid metabolomic data from different growth periods of red and green prickly ash fruit. **(C)** Correlation analysis between samples. **(D)** Heat map of metabolite contents in different growth periods of red and green prickly ash fruit. **(E)** The content and proportion of ten flavonoid classes in different growth periods of red and green prickly ash.

To study the color differences in the two kinds of prickly ash fruit, we focused on the detection of flavonoids (Figure 1A). In the principal component analysis (PCA) of flavonoid content data, the first two principal component axes explained 64.30% of the variation between fruit of different colors and growth periods (PC1 45.40%, PC2 18.90%) (Figure 1B). We performed an intra-group correlation analysis and found that biological replicates of the same fruit color were highly correlated (r^2^ > 0.7), whereas red and green fruit showed large differences (Figure 1C). In addition, there were strong correlations between samples from different growth periods in green prickly ash, indicating that there were few changes in flavonoid content over the course of development in green fruit.

Qualitative and quantitative detection of flavonoids was carried out by LC-MS/MS to compare the differences in flavonoids between two kinds of prickly ash fruit at different growth periods (Figure 1D). LC-MS/MS detected 214 flavonoids, and their distributions differed markedly between red and green fruit (Table S1). Most of the flavonoids were present at high levels in fruit of one color and at low levels in the other. The detected flavonoids were divided into 10 categories (Table S2): chalcones, dihydroflavones, dihydroflavonols, anthocyanins, flavonoids, flavonols, flavonoid carbonosides, flavanols, isoflavones, and proanthocyanidins. The total content of flavonoids increased continuously throughout development in red prickly ash fruit, whereas that of green prickly ash fruit decreased slightly during development (Figure 1E). Flavonoids and flavonols were the main flavonoid components at all growth periods of both kinds of prickly ash fruit. However, anthocyanins were always present at a low level in green prickly ash, whereas they increased significantly during development in red prickly ash.

The content of anthocyanins in red prickly ash fruit was significantly higher than that of other metabolites, whereas anthocyanin content was extremely low during different growth periods of green prickly ash fruit. The main anthocyanins detected were cyanidin-3-O-arabinoside, cyanidin-3-O-glucoside, cyanidin-3-O-galactoside (kuromanin), cyanidin-3-O-rutinoside (keracyanin), petunidin-3-O-glucoside-5-O-arabinoside, and cyanidin-3,5-O-diglucoside (cyanin). Among these, the contents of cyanidin-3-O-glucoside and cyanidin-3-O-galactoside were the highest. Therefore, the major red substances of the red prickly ash fruit during the mature period were anthocyanins.

### Differentially Expressed Genes in Different Growth Periods of Red and Green Prickly Ash

**Figure 2:**
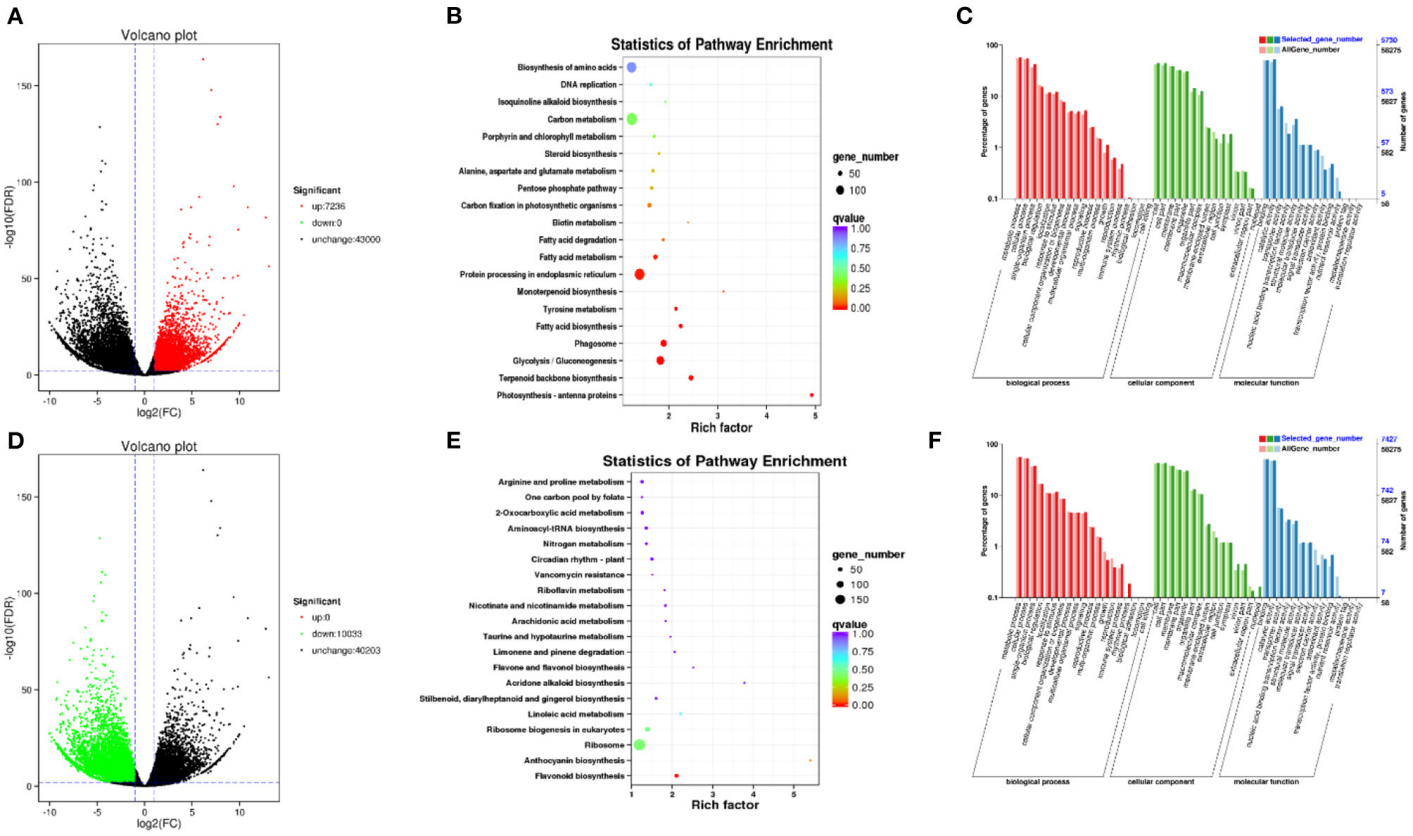
Analysis of differentially expressed genes in red and green prickly ash fruit across all growth periods. **(A)** Volcano plot of genes upregulated in green prickly ash. **(B)** KEGG enrichment analysis of the genes in **(A)**. **(C)** GO enrichment analysis of the genes in **(A)**. **(D)** Volcano plot of genes downregulated in green prickly ash (upregulated in red prickly ash). **(E)** KEGG enrichment analysis of the genes in **(D)**. **(F)** GO enrichment analysis of the genes in **(D)**.

RNA-seq was used to identify DEGs between the two kinds of prickly ash fruit, with a focus on differences in the levels of flavonoid-related genes during fruit development. A total of 64,999 genes were assembled and annotated from the transcriptome data, and Q30 bases accounted for more than 90% of the sequence data in all samples. DEGs were identified based on an adjusted *p*-value of FDR < 0.05 and a fold change ≥2.0. A total of 17,269 DEGs were identified in both kinds of prickly ash fruit across all growth stages (Figures 2A,D): 7,236 upregulated and 10,033 downregulated in green prickly ash compared to red. KEGG pathway and GO enrichment analyses of the DEGs upregulated in green fruit showed that these genes were enriched mainly in biosynthesis of amino acids (ko01230), fatty acid metabolism (ko01212), carbon metabolism (ko01200), protein processing in endoplasmic reticulum (ko04141), and terpenoid backbone biosynthesis (ko00900) (Figures 2B,C). A large number of DEGs were therefore involved in basic life processes during fruit development. The KEGG and GO enrichment analyses of DEGs upregulated in red prickly ash fruit showed that many DEGs were involved in the pathways of flavone and flavonol biosynthesis (ko00944), anthocyanin biosynthesis (ko00942), and flavonoid biosynthesis (ko00941) during red prickly ash fruit development (Figures 2E,F).

**Figure 3:**
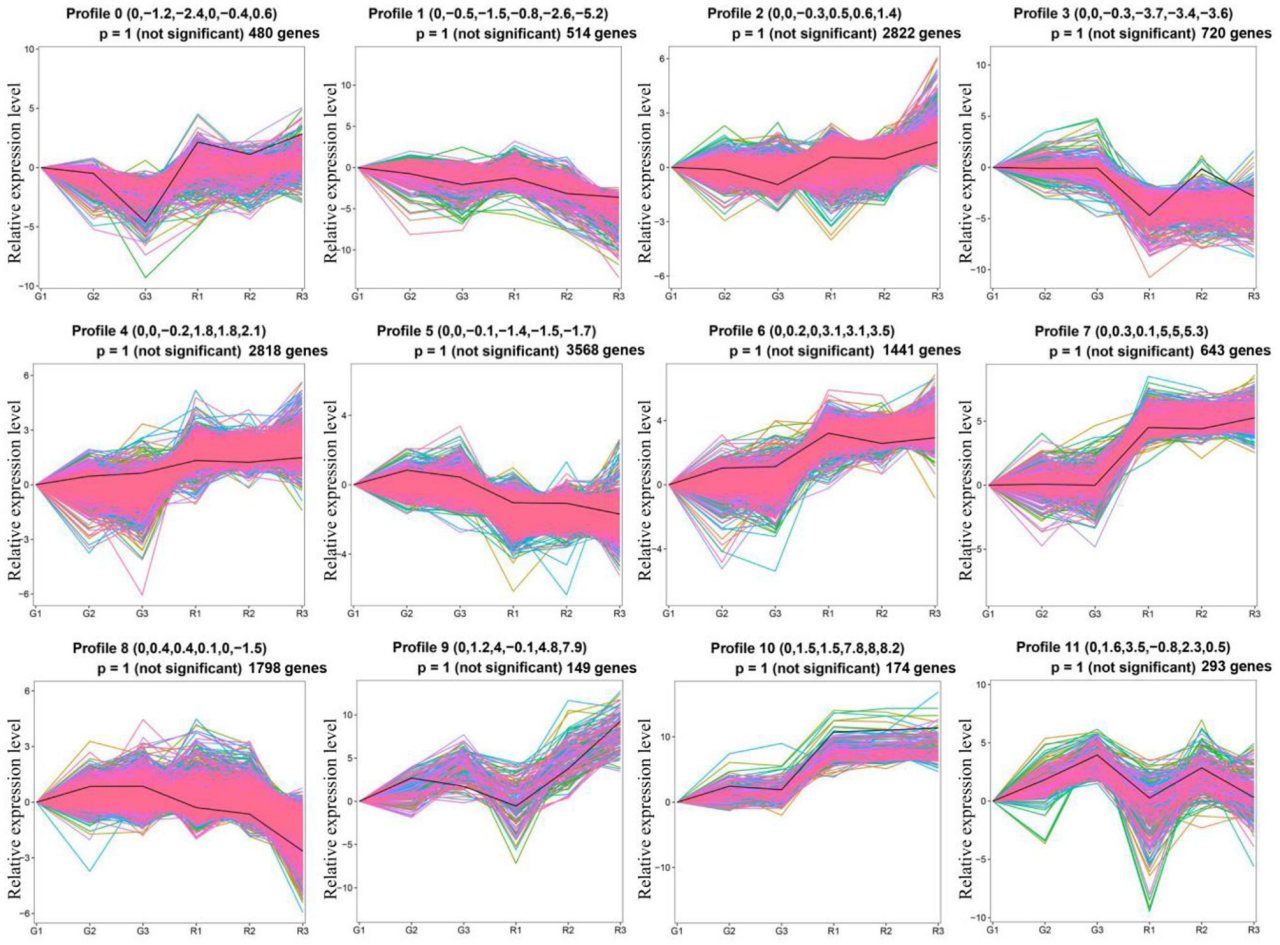
Trend analysis of differentially expressed genes in different growth periods of red and green prickly ash fruit.

To study the gene expression patterns during different growth periods of prickly ash fruit, we performed a trend analysis of the 17,269 DEGs using STEM (Figure 3). The DEGs were divided into twelve profiles (profiles 0–11), which contained 480, 514, 2,822, 720, 2,818, 3,568, 1,441, 643, 1,798, 149, 174, and 293 genes, respectively. More than 1,500 genes were grouped into profiles 2, 4, 5, and 8. Genes in profiles 2 and 4 had higher expression in red prickly ash, whereas genes in profiles 5 and 8 had higher expression in green prickly ash. This result is consistent with the large number of differentially abundant metabolites measured during the growth of both kinds of prickly ash fruit.

### Weighted Gene Co-expression Network Analysis

**Figure 4:**
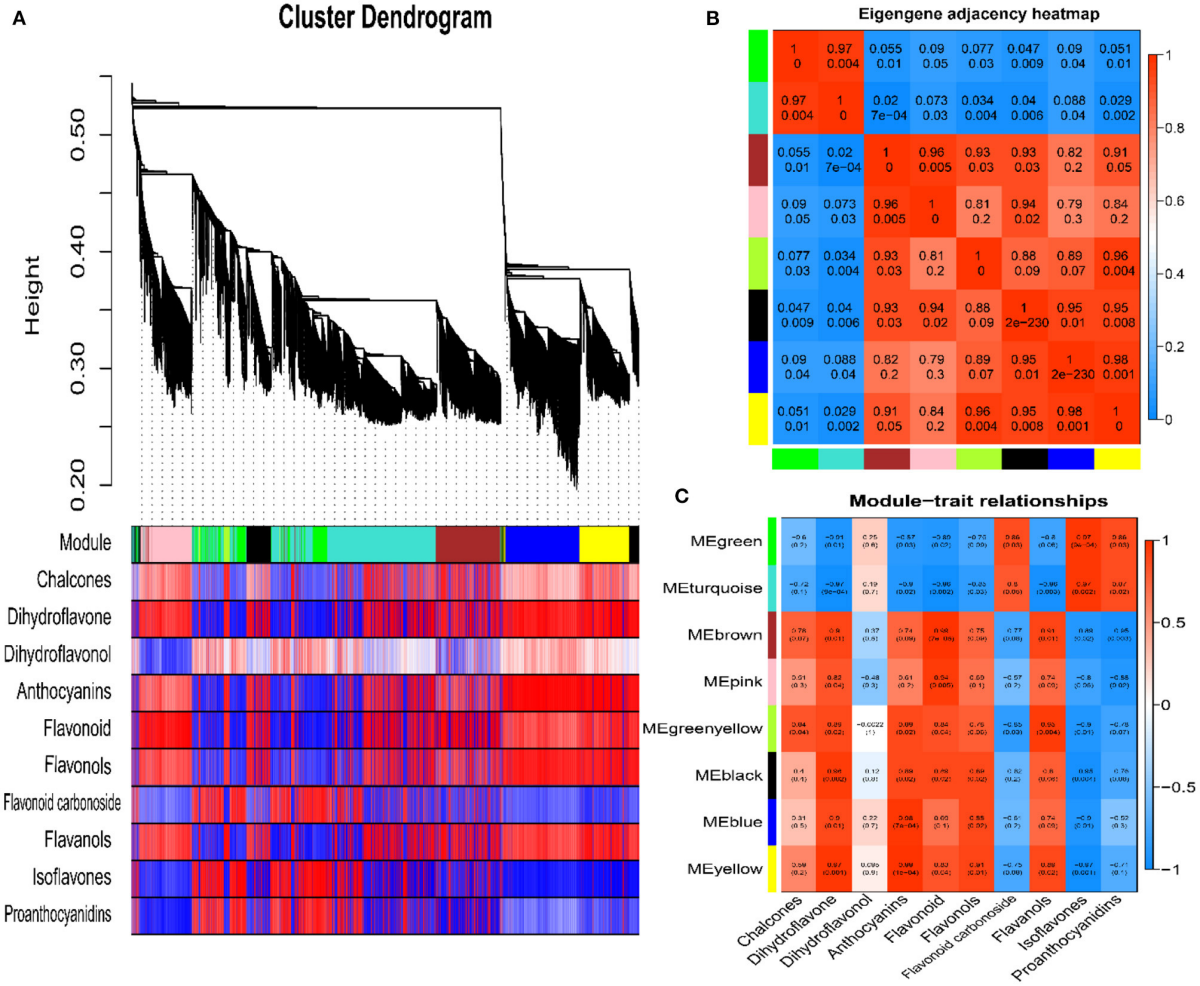
Weighted gene co-expression network analysis. **(A)** Cluster dendrogram of differentially expressed genes. **(B)** Eigengene adjacency heat map. **(C)** Module-trait relationships (upper values are the correlation coefficients, and lower values are the *p*-values).

To study the correlations between genes and metabolites involved in fruit color formation, we performed an association analysis on the content of 10 flavonoid classes and differentially expressed gene modules from red and green fruit using a co-expression network. The WGCNA analysis was based on 12,497 DEGs between red and green fruit at the mature period and the content of chalcones, dihydroflavones, dihydroflavonols, anthocyanins, flavonoids, flavonols, flavonoid carbonosides, flavanols, isoflavones, and proanthocyanidins detected by LC-MS/MS. WGCNA results divided the 12,497 DEGs into eight modules based on their expression patterns: MEgreen, MEturquoise, MEbrown, MEpink, MEgreenyellow, MEblack, MEblue and MEyellow (Figure 4A). The eight modules contained 1,063, 4,244, 1,729, 1,192, 184, 1,001, 1,806, and 1,278 DEGs, respectively.

An eigengene adjacency heat map shows the correlations between different modules (Figure 4B). MEgreen was highly correlated with MEturquoise (r^2^ ≥ 0.97) and negatively correlated with the other modules. The MEbrown, MEpink, MEgreenyellow, MEblack, MEblue, and MEyellow modules were highly correlated with each other (r^2^ ≥ 0.79). The results of metabolome analysis showed that anthocyanins were the main red substances in red prickly ash fruit. The module-trait relationship results showed that anthocyanins were highly correlated with the MEbrown, MEpink, MEgreenyellow, MEblack, MEblue, and MEyellow modules, and they were most highly correlated with the MEbrown module (r^2^ = 0.91, *p* = 0.01) (Figure 4C). To further study the correlation between the MEbrown module and anthocyanin synthesis, we analyzed the 1,729 DEGs in this module. Among them, 37 genes were associated with the flavonoid synthesis pathway, including *DFR, F3'H, LAR, ANR, ANS, UFGT*, and other transcription factor genes related to flavonoid synthesis. Overall, WGCNA can evaluate correlations between phenotypic indicators and gene modules, enabling the study of relationships between phenotype and molecular regulation. In this study, the brown module was highly correlated with anthocyanin levels, providing candidate genes for anthocyanin synthesis, accumulation, and regulation.

### Expression Patterns of Flavonoid Biosynthesis-Related Genes During Prickly Ash Fruit Development

**Figure 5:**
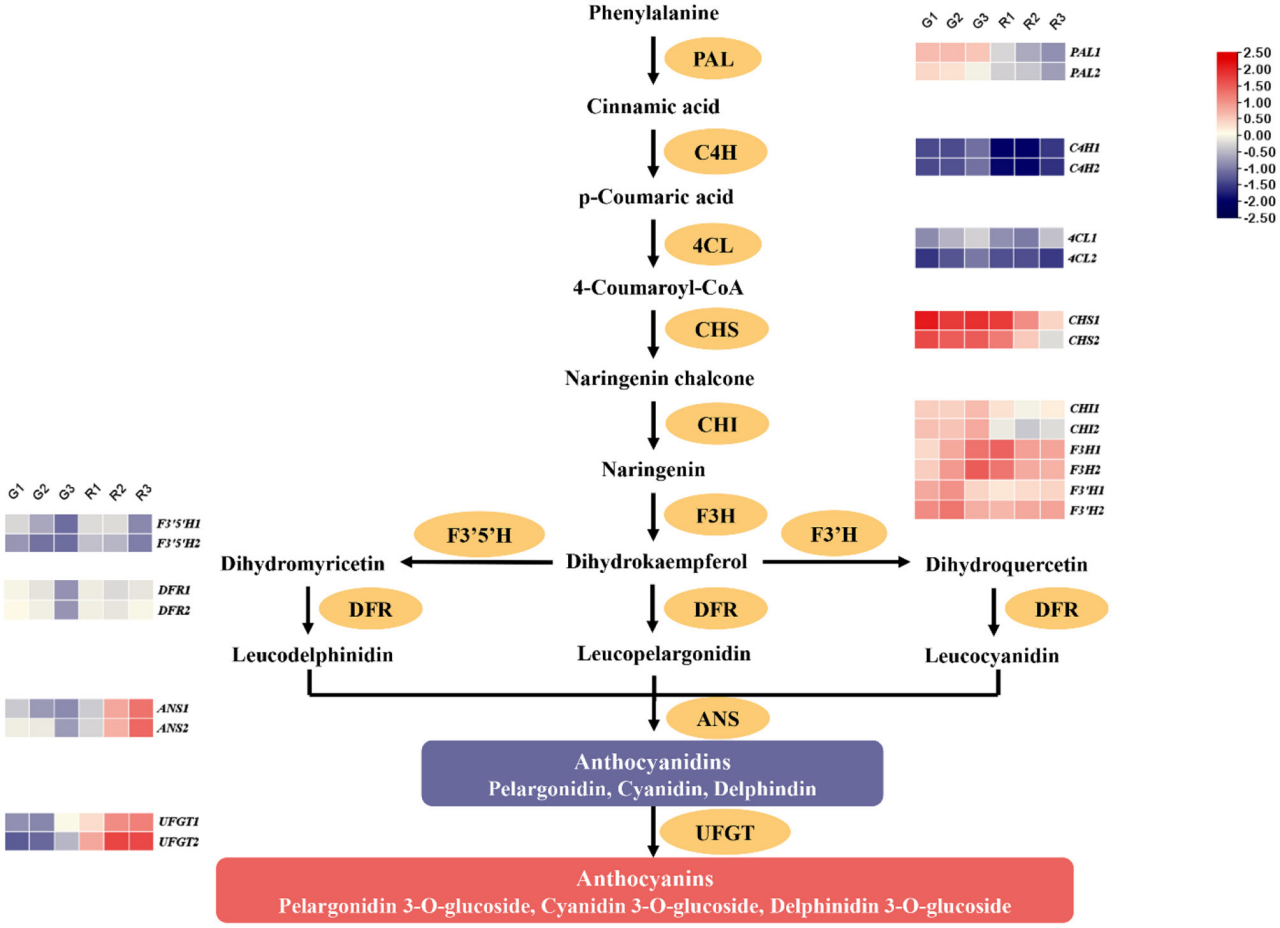
Expression patterns of genes encoding enzymes of the anthocyanin synthesis pathway.

Anthocyanin is the main red component of red prickly ash fruit, and there are significant differences between red and green fruit. The synthesis of anthocyanins occurs mainly by the flavonoid synthesis pathway, and we therefore analyzed gene expression patterns related to flavonoid synthesis during different growth periods of the two kinds of prickly ash fruit (Table S3). Anthocyanin synthesis begins with the conversion of phenylalanine to cinnamic acid catalyzed by PAL. Cinnamic acid is acted upon by C4H and 4CL to form 4-coumaroyl-CoA, which is combined with malonyl-CoA by CHS to form chalcone. CHI catalyzes the formation of flavonoids from chalcone, and the activities of F3H and F3'H produce dihydroflavonols. Subsequently, DFR catalyzes the production of leucoanthocyanidins from dihydroflavonols, and leucoanthocyanidins are finally transformed into colored anthocyanins by ANS. UFGT converts anthocyanidins into various forms of anthocyanin glycoside. *PAL, CHS, CHI, F3'H, F3'5'H*, and *DFR* genes act upstream in the flavonoid biosynthesis pathway, and their expression levels decreased in both red and green prickly ash fruit during development (Figure 5). However, *4CL, F3H, ANS*, and *UFGT* genes showed opposite expression patterns in red and green fruit. The expression levels of these genes were low throughout development of green prickly ash but high during development of red prickly ash. *ANS* and *UFGT* function in the later stages of anthocyanin synthesis, and expression of their genes continued to rise during red prickly ash fruit development, consistent with the measured trend in anthocyanin content. The expression models of flavonoid synthesis genes appeared to explain the differences in flavonoid content between the two prickly ash fruit types, and the *ANS* and *UFGT* genes played a significant role in anthocyanin synthesis in red prickly ash fruit.

### Canonical Correlation Analysis

**Figure 6:**
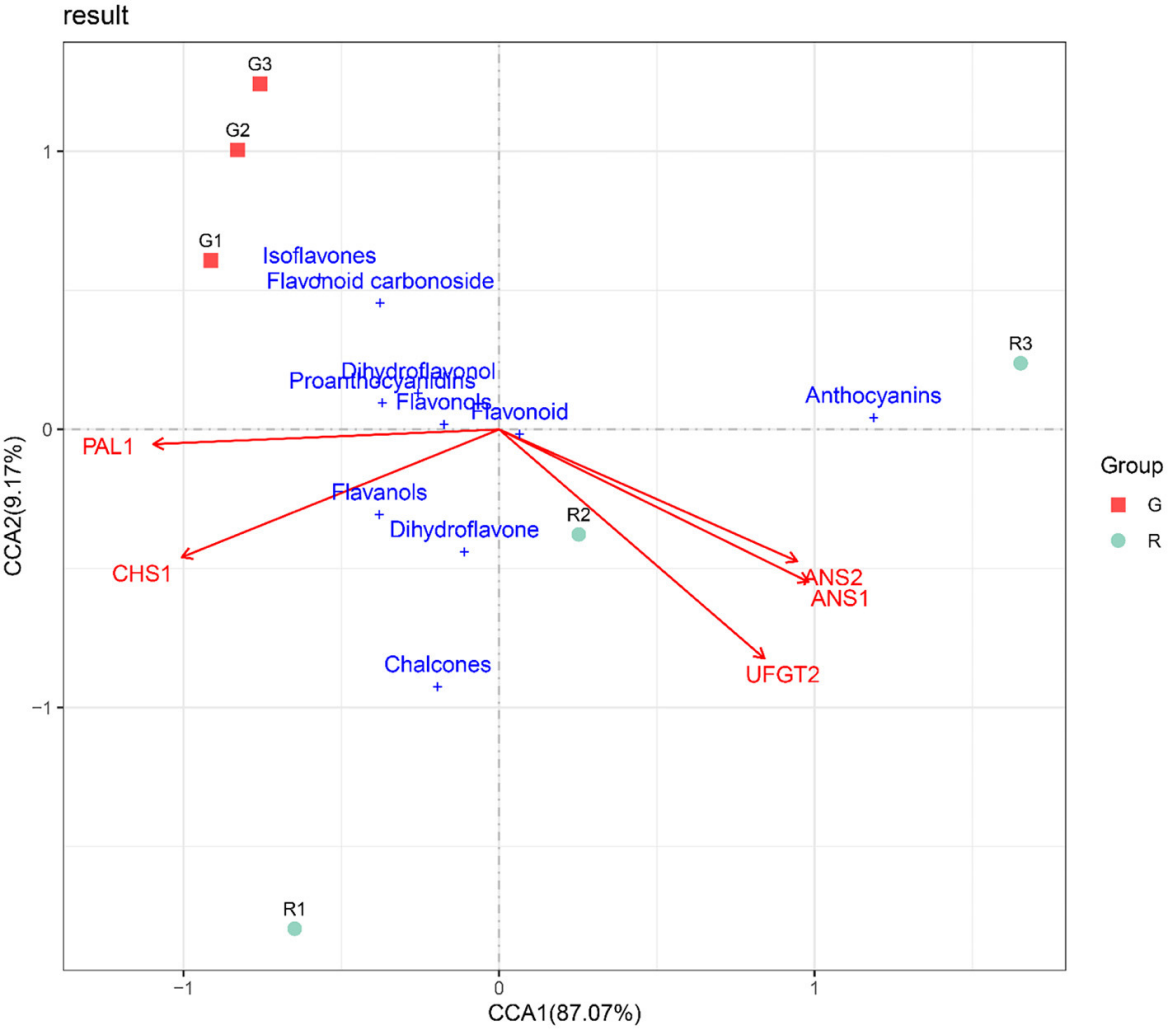
RDA analysis of flavonoid synthesis-related genes and flavonoid content in different growth periods of red and green prickly ash fruit.

To identify the key anthocyanin synthesis genes in the two kinds of prickly ash fruit, we performed canonical correlation analysis on flavonoid contents and expression levels of flavonoid synthesis genes in the two kinds of fruit at different developmental stages. The CCA results indicated that the two fruit types differed markedly in the synthesis and accumulation of flavonoids. CCA1 and CCA2 explained 87.07 and 9.17% of the relationship between flavonoid content and gene expression levels of the flavonoid biosynthesis pathway in different developmental stages of red and green prickly ash fruit, indicating that the CCA results could fully demonstrate the relationship between the response variables (flavonoid contents) and the explanatory variables (gene expression levels). In addition, *ANS1, ANS2*, and *UFGT* genes were positively correlated with the contents of anthocyanin and flavonoids but negatively correlated with isoflavones and flavonoid carbonosides (Figure 6). The distribution of anthocyanins was close to that of the red prickly ash samples, indicating that their content in red prickly ash was higher. In general, anthocyanins were the essential flavonoid components of red prickly ash, and *ANS1, ANS2*, and *UFGT* were the key genes for anthocyanin synthesis.

## Discussion

Anthocyanins have created a rich and colorful plant kingdom. At present, more than 20 kinds of anthocyanin have been found in nature (30), six of which are widely distributed in plant tissues and organs. These include delphinidin (Dp), pelargonidin (Pg), malvidin (Mv), petunidin (Pt), peonidin (Pn), and cyanidin (Cy) (20). Different types of anthocyanin have different colors. For example, the pigment of geranium is orange-red, the pigment of chamomile is cardinal red, and the pigment of oat grass is blue-violet (31). Anthocyanins have higher antioxidant activity than other flavonoids, and their antioxidant activity depends on the type of B ring group they possess. The antioxidant capacities of anthocyanins have been ranked Pg < Pn < Cy = Mv < Pt < Dp (32). The study of anthocyanin synthesis mechanisms has promoted improvements in fruit, flower, and vegetable quality. Research on kiwifruit (33, 34), apple (35), jujube (36), litchi (37), grape (38, 39), and other plants has contributed to our understanding of anthocyanin synthesis mechanisms of economically important woody species, which is important for cultivating elite fruit tree varieties that are rich in anthocyanin.

Anthocyanins, as water-soluble flavonoid pigments, can give a variety of colors to plants and have nutritional and health-promoting functions in the human body. They have anti-aging properties and can help to prevent cardiovascular diseases and improve blood sugar and eyesight. Plants rich in anthocyanins can be developed as functional foods and clinical drugs. Different plants have different anthocyanin types. The major anthocyanins in mulberry fruit are cyanidin-3-glucoside and cyanidin-3-rutinoside (40), whereas the major anthocyanins in pomegranate are cyanidin-3-glucoside and delphinidin-3-glucoside (41). Cyanidin-3-O-rutinoside, cyanidin, and paeoniflorin-3,5-O-diglucoside are the major red components of jujube peel (42). Sixteen anthocyanins were identified in purple and green asparagus (*Asparagus officinalis*) by high performance liquid chromatography, and paeoniflorin, glycoside derivatives, and cyanidin were the main components (43).

Here, we used transcriptomics and metabolomics to identify differentially expressed genes and metabolites in different growth periods of red and green fruit. We identified 17,269 differentially expressed genes and detected 214 flavonoids. Among them, green peppercorns up-regulated 7,236 genes, while red peppercorns up-regulated 10,033 genes significantly higher than green peppercorns. The enrichment analysis of the differential genes showed that during fruit development, prickly ash mainly focused on the synthesis of primary metabolites, such as fatty acid synthesis, protein processing and glycolysis. While red pepper fruit development has a large number of genes involved in the synthesis of secondary metabolites, such as the biosynthesis of flavonoids and anthocyanin biosynthesis. In addition, the total amount of metabolites in red prickly ash was significantly higher than that in green prickly ash, mainly due to the high content of anthocyanin in red prickly ash and almost no anthocyanin in green prickly ash. Using WGCNA analysis and the comparison of color components, we identified anthocyanins as the main metabolite responsible for the color differences between red and green prickly ash fruit. Based on the flavonoid synthesis pathway combined with metabolite content and gene expression data, we determined that *ANS* and *UFGT* were the key genes responsible for enhanced anthocyanin synthesis of red prickly ash fruit, and cyanidin-3-O-glucoside and cyanidin-3-O-galactoside were the major anthocyanin constituents.

With continued research on plant pigments, the synthesis and regulatory mechanisms of flavonoids and anthocyanins have gradually been clarified. The synthesis of anthocyanins is achieved mainly through the flavonoid synthesis pathway. The pathway takes phenylalanine as its initial substrate and forms anthocyanins through the catalysis of PAL, 4CL, C4H, CHI, CHS, F3'H, F3H, ANS, DFR, and UFGT. Anthocyanidin synthase (ANS/LDOX) is the main enzyme at the end of the anthocyanin synthesis pathway and catalyzes the major reaction that converts leucocyanidin into colored anthocyanidin. UFGT can catalyze the glycosylation of anthocyanidins at positions 3 and 5 to convert unstable anthocyanidins into stable anthocyanins. Anthocyanin-free cultivars of sweet orange cannot synthesize anthocyanins due to a lack of UFGT, indicating that UFGT is an indispensable enzyme for anthocyanin synthesis (44). *ANS* and *UFGT* were shown to be key genes for anthocyanin synthesis during the growth of red prickly ash fruit. They had high expression levels in all three growth periods of red prickly ash, but they were minimally expressed in green prickly ash. Transcription factors from the bHLH, MYB, and WD40 families can also control the synthesis of anthocyanins. MYB transcription factors typically combine with WD40 and bHLH transcription factors to produce a ternary MYB-bHLH-WD40 complex that regulates anthocyanin synthesis. The synthesis of anthocyanins is also affected by environmental factors, and light can promote the synthesis of anthocyanins in plants. In addition, the accumulation of anthocyanins can also increase in response to high temperatures, UV light, drought and nutrient deficiencies (45). The results of this study revealed the basis for the color difference between red and green prickly ash fruit, clarified the mechanism of anthocyanin synthesis in prickly ash, and provided a basis for the development of foods and medicines from prickly ash.

## Data Availability Statement

The datasets presented in this study can be found in an online repository. The name of the repository and accession number can be found below: https://www.ncbi.nlm.nih.gov/search/all/?term=PRJNA756714.

## Author Contributions

XF: methodology, validation, data curation, writing—original draft preparation, writing—review, and editing. YW: formal analysis, software, writing—review, and editing. YQ: conceptualization, software, and data curation. HH and YL: validation. AW: conceptualization and project administration. All authors have read and agreed to the published version of the manuscript. All authors contributed to the article and approved the submitted version.

## Funding

This study was financially supported by the Technology Innovation Guidance Special Foundation of Shaanxi Province (2020QFY07-01).

## Conflict of Interest

The authors declare that the research was conducted in the absence of any commercial or financial relationships that could be construed as a potential conflict of interest.

## Publisher's Note

All claims expressed in this article are solely those of the authors and do not necessarily represent those of their affiliated organizations, or those of the publisher, the editors and the reviewers. Any product that may be evaluated in this article, or claim that may be made by its manufacturer, is not guaranteed or endorsed by the publisher.
