# Supplementary material for: Integrated LC-MS/MS and Transcriptome Sequencing Analysis Reveals the Mechanism of Color Formation During Prickly Ash Fruit Ripening
Source: Front Nutr. 2022 Mar 16;9:847823. doi: 10.3389/fnut.2022.847823 (PMC8967253; doi:10.3389/fnut.2022.847823)
Supplement: Supplementary file 1 [file Table_1.DOCX]

**Table S1.** The content of flavonoids in different growth periods of red and green prickly ash fruit.

| **Compounds** | **Class** | **R1** | **R2** | **R3** | **G1** | **G2** | **G3** |
| --- | --- | --- | --- | --- | --- | --- | --- |
| Naringenin chalcone | Chalcones | 115549 | 43399.67 | 20463.43 | 96999.33 | 51004 | 30683.67 |
| Phloretin | Chalcones | 33782.9 | 8724.5 | 6896.3 | 28227 | 23097.67 | 25873 |
| Xanthohumol | Chalcones | 53688.67 | 34703.33 | 19863.1 | 2963.8 | 3434.867 | 3561.5 |
| Isosalipurposide (Phlorizin Chalcone) | Chalcones | 255896.7 | 40117.33 | 15262.17 | 247196.7 | 89613.33 | 52183 |
| Phloretin-2'-O-glucoside (Phlorizin) | Chalcones | 2891707 | 1030077 | 749386.7 | 41650.67 | 25473 | 19994.67 |
| Pinocembrin (Dihydrochrysin) | Dihydroflavone | 36373 | 48030.67 | 50669.67 | 60105.33 | 57903 | 56901.33 |
| Naringenin (5,7,4'-Trihydroxyflavanone) | Dihydroflavone | 303420 | 609573.3 | 403823.3 | 875336.7 | 858150 | 667293.3 |
| Eriodictyol (5,7,3',4'-Tetrahydroxyflavanone) | Dihydroflavone | 48288 | 73217 | 46779.33 | 51408 | 42796.67 | 36675 |
| Hesperetin | Dihydroflavone | 9709.633 | 32171.83 | 22389 | 6480.7 | 11012.23 | 11399.97 |
| Persicogenin (5,3'-dihydroxy-7,4'-dimethoxyflavanone) | Dihydroflavone | 22631.67 | 2281.4 | 6280.567 | 10945.77 | 29947.17 | 22880.83 |
| Naringenin-7-O-glucoside (Prunin) | Dihydroflavone | 446680 | 147633.3 | 156723.3 | 412063.3 | 330683.3 | 241283.3 |
| Eriodictyol-8-C-glucoside | Dihydroflavone | 912676.7 | 698916.7 | 515860 | 440983.3 | 302800 | 170906.7 |
| Persicoside | Dihydroflavone | 1086750 | 1536410 | 2725333 | 285256.7 | 389413.3 | 510720 |
| Naringenin-7-O-Rutinoside(Narirutin)* | Dihydroflavone | 67552.67 | 99494 | 150550.7 | 60828.33 | 44124.67 | 33886.33 |
| Naringenin-7-O-Neohesperidoside(Naringin)* | Dihydroflavone | 108348 | 130985 | 165287.3 | 68615 | 61844.67 | 48268.33 |
| Poncirin (Isosakuranetin-7-O-neohesperidoside) | Dihydroflavone | 188622.3 | 706027.7 | 937050 | 23272.33 | 17903 | 18895 |
| Neoponcirin | Dihydroflavone | 11407.27 | 33278.27 | 30506.7 | 9 | 9 | 9 |
| Hesperetin-7-O-neohesperidoside(Neohesperidin) | Dihydroflavone | 7236800 | 3280867 | 1753000 | 1775000 | 1063127 | 569603.3 |
| Hesperetin-7-O-rutinoside (Hesperidin) | Dihydroflavone | 7534933 | 3510167 | 1837900 | 1846600 | 1078773 | 623030 |
| Pinobanksin | Dihydroflavonol | 113717.7 | 33387.33 | 13073.6 | 85114 | 38947.67 | 23743.67 |
| Aromadendrin (Dihydrokaempferol) | Dihydroflavonol | 23513.23 | 27866.3 | 13410.47 | 95684.33 | 133370 | 133400 |
| Fustin | Dihydroflavonol | 568713.3 | 354586.7 | 249500 | 616466.7 | 581926.7 | 429760 |
| Dihydroquercetin(Taxifolin) | Dihydroflavonol | 140913.3 | 33171.33 | 12680.9 | 96250.33 | 84203.33 | 46075 |
| 3-O-Acetylpinobanksin | Dihydroflavonol | 92688 | 46607.33 | 41699.33 | 31482.67 | 131976.7 | 147693.3 |
| Taxifolin-3-O-rhamnoside (Astilbin) | Dihydroflavonol | 71826.67 | 63471.33 | 78939.67 | 162923.3 | 235506.7 | 284340 |
| Hesperetin-5-O-glucoside | Dihydroflavonol | 1530967 | 1729333 | 1666400 | 1382300 | 1315867 | 1048267 |
| Cyanidin-3-O-arabinoside | Anthocyanins | 2912300 | 25174333 | 56580333 | 9 | 9 | 9 |
| Cyanidin-3-O-galactoside* | Anthocyanins | 2443367 | 21387500 | 60270667 | 9 | 9 | 9 |
| Cyanidin-3-O-glucoside (Kuromanin)* | Anthocyanins | 2580433 | 21024900 | 58380000 | 9 | 9 | 9 |
| Cyanidin-3-O-rutinoside (Keracyanin) | Anthocyanins | 3109400 | 11217200 | 66304333 | 1254233 | 754823.3 | 808476.7 |
| Petunidin-3-O-glucoside-5-O-arabinoside | Anthocyanins | 421460 | 188060 | 105450 | 1135037 | 553586.7 | 307210 |
| Cyanidin-3,5-O-diglucoside (Cyanin) | Anthocyanins | 751573.3 | 328836.7 | 205553 | 1787000 | 866840 | 492223.3 |
| Tectochrysin | Flavonoid | 2332.633 | 2369.833 | 3262.133 | 9 | 9 | 9 |
| 5,7,8-Tetrahydroxy-6-methoxyflavone | Flavonoid | 225186.7 | 831200 | 1012507 | 6588 | 6868.8 | 7323.067 |
| Diosmetin (5,7,3'-Trihydroxy-4'-methoxyflavone) | Flavonoid | 108302.3 | 120564.3 | 128515.7 | 13418 | 14133.5 | 27107 |
| Tricetin (5,7,3',4',5'-Pentahydroxyflavone) | Flavonoid | 9 | 9 | 9 | 60311 | 9983.7 | 9 |
| Dihydroxy-dimethoxyflavone | Flavonoid | 101619.1 | 32787.67 | 91519.33 | 2630.7 | 44312.33 | 106336.3 |
| Tamarixetin (3,3',5,7-Tetrahydroxy-4'-Methoxyflavone) | Flavonoid | 199800 | 347926.7 | 340800 | 419113.3 | 514106.7 | 409010 |
| Quercetagetin-3-Methyl Ether* | Flavonoid | 316376.7 | 525650 | 452626.7 | 157753 | 180670 | 150816.7 |
| Quercetagetin-4'-Methyl Ether* | Flavonoid | 600993.3 | 794396.7 | 790070 | 232970 | 223270 | 183320 |
| 5,6,7,4'-Tetramethoxyflavone | Flavonoid | 8397.333 | 5484.967 | 3861.567 | 6627.467 | 6970.667 | 5365.033 |
| Eupatilin (5,7-Dihydroxy-3',4',6-Trimethoxyflavone) | Flavonoid | 88641.37 | 41975.33 | 127504.7 | 9 | 16080.67 | 27719 |
| Penduletin (5,4'-Dihydroxy-3,6,7-trimethoxyflavone) | Flavonoid | 118875.6 | 37255 | 38359 | 3689.333 | 31633.67 | 49487.3 |
| 5,7-Dihydroxy-3',4',5'-trimethoxyflavone | Flavonoid | 197457.4 | 63051.33 | 69399.83 | 4900 | 51222.2 | 81669 |
| 5,6,7,4'-Tetramethoxyflavanone | Flavonoid | 36981.47 | 13233.13 | 15970.67 | 9 | 11493.4 | 22479 |
| Limocitrin (5,7,4'-trihydroxy-8,3'-dimethoxyflavone) | Flavonoid | 6728700 | 12251000 | 12901633 | 7724900 | 11139333 | 10702033 |
| Syringetin | Flavonoid | 332843.3 | 543003.3 | 453056.7 | 398803.3 | 514420 | 543913.3 |
| Quercetagetin-3,4'-Dimethyl Ether | Flavonoid | 172297 | 338280 | 355256.7 | 274660 | 386200 | 397070 |
| Centaureidin (5,7,3'-Trihydroxy-3,6,4'-Trimethoxyflavone) | Flavonoid | 11634.13 | 6542.3 | 7813.033 | 4603.9 | 2074.467 | 5071.833 |
| 4',5,7-Trihydroxy-3',3,6-Trimethoxyflavone (Jaceidin) | Flavonoid | 45830.67 | 6786.603 | 14577.37 | 16106.13 | 10318.23 | 20188.43 |
| 5-Hydroxy-6,7,8,3',4'-pentamethoxyflavone | Flavonoid | 3264.367 | 2407.3 | 1730.033 | 3898.7 | 5232.4 | 3920.033 |
| Apigenin-8-C-Arabinoside | Flavonoid | 6833.033 | 10714.83 | 10636.77 | 23373.33 | 21354.33 | 18727.33 |
| Nobiletin (5,6,7,8,3',4'-Hexamethoxyflavone) | Flavonoid | 227686.7 | 165606.7 | 155116.7 | 349770 | 330616.7 | 329230 |
| Kaempferol-3-O-arabinoside | Flavonoid | 6458667 | 2718280 | 1845127 | 9 | 9 | 9 |
| Loquatoside | Flavonoid | 1911.1 | 16569.67 | 16503.17 | 14365.33 | 47164 | 93545.33 |
| Acacetin-7-O-galactoside | Flavonoid | 36117.67 | 10575.67 | 13676.6 | 9 | 9 | 9 |
| Kaempferol-4'-O-glucoside | Flavonoid | 16102000 | 10271300 | 9272967 | 39933000 | 36882667 | 34702333 |
| Aromadendrin-7-O-glucoside | Flavonoid | 2567267 | 1739600 | 1269267 | 1055067 | 716776.7 | 367956.7 |
| Luteolin-7-O-glucuronide | Flavonoid | 56476.83 | 38034.5 | 20125 | 84720.67 | 76896.67 | 56961.67 |
| Tetahydroxyflavone-7-O-glucuronide | Flavonoid | 30977.33 | 46106.67 | 46178 | 61110 | 73819.33 | 64842.67 |
| Pratensein-7-O-glucoside | Flavonoid | 8166400 | 27528333 | 26061333 | 201380 | 269216.7 | 307733.3 |
| Chrysoeriol-7-O-glucoside | Flavonoid | 571046.7 | 2312633 | 1306503 | 1686067 | 3217800 | 2523233 |
| Chrysoeriol-5-O-glucoside | Flavonoid | 5690300 | 7324833 | 7078367 | 6263733 | 6640900 | 5156300 |
| Dihydroxy-dimethoxyflavone-7-O-glucoside | Flavonoid | 7954 | 33872 | 9 | 34288 | 100447.3 | 78937 |
| Quercetagetin-7-O-glucoside | Flavonoid | 15148667 | 8146400 | 4878700 | 1204233 | 677460 | 406266.7 |
| Tricin-7-O-Glucoside | Flavonoid | 283200 | 36449.33 | 9 | 1919100 | 2371100 | 1637967 |
| Tricin-5-O-Glucoside | Flavonoid | 36723 | 147093.7 | 248413.3 | 9 | 9 | 9 |
| Laricitrin-3-O-glucoside | Flavonoid | 296337 | 9 | 9 | 9 | 9 | 9 |
| Mearnsetin-3-O-glucoside | Flavonoid | 7362177 | 21692467 | 35885667 | 9931267 | 8661833 | 10818633 |
| Chrysoeriol-7-O-(6''-acetyl)glucoside | Flavonoid | 428196.7 | 524990 | 469450 | 1159967 | 1041433 | 752376.7 |
| Limocitrin-3-O-galactoside | Flavonoid | 14991200 | 27423000 | 32553667 | 7439800 | 9361933 | 10792000 |
| Syringetin-7-O-glucoside* | Flavonoid | 15854100 | 29083333 | 33450333 | 7389900 | 9148067 | 10727667 |
| Syringetin-3-O-glucoside* | Flavonoid | 1090067 | 1971900 | 2094233 | 1516100 | 2647433 | 3296700 |
| Tricin-7-O-saccharic acid | Flavonoid | 101919.3 | 40150 | 21589.33 | 831846.7 | 795580 | 627630 |
| Laricitrin-3-O-(6''-acetyl)glucoside | Flavonoid | 551663.3 | 600193.3 | 514756.7 | 162586.7 | 122749.7 | 164963.3 |
| Diosmetin-7-O-(6''-malonyl)glucoside | Flavonoid | 34641.33 | 22801.67 | 16586 | 13012 | 11309.07 | 10982.83 |
| Kaempferide-3-O-(6''-malonyl)glucoside | Flavonoid | 1133397 | 1759350 | 1977817 | 411136.7 | 509856.7 | 666500 |
| Chrysoeriol-7-O-(6''-malonyl)glucoside | Flavonoid | 387486.7 | 243250 | 166306.7 | 138463.3 | 135630 | 118886.7 |
| Syringetin-3-O-(6''-Acetyl)glucoside | Flavonoid | 1998827 | 1539180 | 1219333 | 99224.33 | 104596.7 | 98574 |
| Isorhamnetin-3-O-(6''-malonylglucoside) | Flavonoid | 3595500 | 2916167 | 1860967 | 1507733 | 1364133 | 1202300 |
| Tamarixetin-3-O-(6''-malonyl)glucoside | Flavonoid | 2946700 | 2368400 | 1501167 | 1165600 | 1117933 | 1005057 |
| Tricin-7-O-(6''-Malonyl)Glucoside | Flavonoid | 1043137 | 489360 | 409456.7 | 1491500 | 1412433 | 1374333 |
| Apigenin-7-O-(6''-p-Coumaryl)glucoside | Flavonoid | 928720 | 681100 | 518863.3 | 1689800 | 1237933 | 967863.3 |
| Apigenin-7-O-rutinoside (Isorhoifolin) | Flavonoid | 775280 | 571936.7 | 471236.7 | 1442933 | 1081267 | 887083.3 |
| Luteolin-7-O-glucoside-5-O-arabinoside | Flavonoid | 603230 | 397063.3 | 287409.7 | 80822.33 | 61894.67 | 45279 |
| Chrysoeriol-6-C-rhamnoside-7-O-rhamnoside | Flavonoid | 812140 | 987566.7 | 798720 | 5488.267 | 4227.267 | 4011.867 |
| Acacetin-7-O-rutinoside (Linarin) | Flavonoid | 1624430 | 2006280 | 1658937 | 10520.83 | 7059.5 | 5897.2 |
| Luteolin-7-O-neohesperidoside (Lonicerin) | Flavonoid | 2317833 | 1445700 | 1108507 | 1076000 | 771766.7 | 666823.3 |
| Luteolin-7-O-rutinoside | Flavonoid | 2875433 | 1910800 | 1341367 | 1103400 | 820736.7 | 636046.7 |
| Apigenin-6,8-di-C-glucoside (Vicenin-2) | Flavonoid | 904150 | 1381293 | 1092290 | 1257487 | 1314967 | 876550 |
| Diosmetin-7-O-Neohesperidoside (Neodiosmin) | Flavonoid | 35370667 | 20187333 | 14768667 | 3473433 | 2302567 | 1724800 |
| Chrysoeriol-7-O-rutinoside | Flavonoid | 6185533 | 3491533 | 2329533 | 968393.3 | 715020 | 416193.3 |
| Diosmetin-7-O-rutinoside (Diosmin) | Flavonoid | 4917067 | 2459133 | 1701600 | 374566.7 | 265900 | 202100 |
| Luteolin-6-C-(2''-glucuronyl)glucoside | Flavonoid | 9 | 9 | 9 | 1830.333 | 114239 | 98954.67 |
| Tamarixetin-3-O-rutinoside | Flavonoid | 9141000 | 14792667 | 16833667 | 455666.7 | 362186.7 | 232286.7 |
| Chrysoeriol-5,7-di-O-glucoside | Flavonoid | 46183.33 | 266570 | 978706.7 | 34369.67 | 9 | 42643.67 |
| Chrysoeriol-6-C-glucoside-4'-O-glucoside | Flavonoid | 29675 | 128604 | 420353.3 | 9 | 9 | 35635.33 |
| Tricin-4'-O-glucoside-7-O-glucoside | Flavonoid | 91766.67 | 332800 | 673540 | 29656 | 26641.33 | 46213.67 |
| Tricin-7-O-(2''-O-glucosyl)glucoside | Flavonoid | 156590.7 | 341476.7 | 648396.7 | 68297.67 | 98939.67 | 107385 |
| Tricin-4'-O-(syringyl alcohol)ether-7-O-glucoside | Flavonoid | 11791.83 | 38198 | 58835.9 | 9 | 9 | 9 |
| Sudachiin B | Flavonoid | 2416450 | 7441450 | 10113163 | 9 | 9 | 9 |
| Sudachiin C | Flavonoid | 2575690 | 7735590 | 10865220 | 9 | 9 | 9 |
| Chrysoeriol-7-O-(6''-sinapoyl)glucoside | Flavonoid | 9 | 9 | 9 | 33423 | 26677.67 | 21510.67 |
| Limocitrin-3,7-di-O-glucoside | Flavonoid | 3398600 | 6395133 | 9281000 | 3858600 | 6865033 | 11102133 |
| Limocitrin-3-O-sophoroside | Flavonoid | 3341900 | 6546733 | 9061700 | 3762167 | 7217833 | 10627567 |
| Tricin-7-O-(2''-Sinapoyl)glucoside | Flavonoid | 4329.433 | 4884.267 | 2599.633 | 9 | 10478.3 | 9343.6 |
| Tricin-4'-O-oxalic acid-7-O-(p-coumaroyl)shikimic acid | Flavonoid | 9 | 1799.7 | 2686.8 | 8047.9 | 9779.433 | 11967.33 |
| Apigenin-7-O-glucoside-4'-O-rutinoside | Flavonoid | 17981 | 29716.33 | 25407 | 9 | 9 | 9 |
| Naringenin-7-O-Rutinoside-4'-O-glucoside | Flavonoid | 17892.67 | 18510.33 | 17581 | 9 | 9 | 9 |
| Isovitexin-2''-O-(6'''-feruloyl)glucoside | Flavonoid | 19389.67 | 47837.33 | 50919 | 9 | 9 | 9 |
| Chrysoeriol-7-O-rutinoside-5-O-glucoside | Flavonoid | 214280.3 | 637086.7 | 433296.7 | 68744.67 | 47648 | 26697 |
| Kaempferol-6,8-di-C-glucoside-7-O-glucoside | Flavonoid | 78099 | 65533.67 | 62478 | 133723.3 | 99083 | 102645 |
| Chrysoeriol-6,8-di-C-glucoside-4'-O-glucoside | Flavonoid | 112678 | 167493.3 | 176533.3 | 28013 | 34803.33 | 24335.67 |
| Tricin-7-O-(2''-feruloyl)glucoside-5-O-glucoside | Flavonoid | 9 | 9 | 9 | 42681.33 | 30155 | 24416.67 |
| Kaempferol (3,5,7,4'-Tetrahydroxyflavone) | Flavonols | 21240.73 | 20487.63 | 18698 | 111067 | 111561.7 | 99599 |
| Herbacetin | Flavonols | 13521.87 | 7678.067 | 3710.367 | 3154.57 | 3451.233 | 2355.367 |
| Azaleatin (5-O-Methylquercetin) | Flavonols | 1086507 | 884336.7 | 9 | 801640 | 625133.3 | 589890 |
| Isorhamnetin | Flavonols | 727570 | 1425770 | 1533767 | 1623700 | 1897600 | 1526600 |
| Gossypetin(3,3',4',5,7,8-Hexahydroxyflavone) | Flavonols | 9 | 9 | 9 | 43045.33 | 45031.33 | 39494.33 |
| 3,7-Di-O-methylquercetin | Flavonols | 9 | 170523.3 | 669643.3 | 59964.33 | 104953.7 | 124979.3 |
| Patuletin (Quercetagetin-6-methyl ether) | Flavonols | 2684100 | 3512033 | 3398433 | 1034010 | 995066.7 | 802470 |
| Tangeretin (4',5,6,7,8-Pentamethoxyflavone) | Flavonols | 294536.7 | 248243.3 | 220486.7 | 332766.7 | 313896.7 | 318246.7 |
| Kaempferol-3-O-arabinoside (Juglanin) | Flavonols | 2116933 | 825656.7 | 548903.3 | 9 | 9 | 9 |
| Kaempferol-3-O-rhamnoside (Afzelin)(Kaempferin)* | Flavonols | 2261227 | 1096587 | 773036.7 | 9 | 9 | 9 |
| Kaempferol-7-O-rhamnoside* | Flavonols | 2358007 | 1071473 | 788833.3 | 9 | 9 | 9 |
| Avicularin(Quercetin-3-O-α-L-arabinofuranoside) | Flavonols | 3951500 | 3722133 | 3413067 | 9 | 9 | 9 |
| Quercetin-3-O-xyloside (Reynoutrin) | Flavonols | 16614000 | 11415000 | 7592167 | 5326567 | 4562033 | 3648000 |
| Kaempferol-3-O-galactoside (Trifolin) | Flavonols | 792756.7 | 595143.3 | 733636.7 | 1400867 | 1304933 | 1085273 |
| Quercetin-3-O-rhamnoside(Quercitrin) | Flavonols | 13171733 | 11711933 | 10926633 | 9 | 9 | 9 |
| Myricetin-3-O-arabinoside | Flavonols | 3942633 | 2454500 | 1300353 | 9 | 9 | 9 |
| 8-Methoxykaempferol-7-O-rhamnoside | Flavonols | 378783.3 | 187125 | 127457.7 | 9 | 9 | 9 |
| Quercetin-3-O-galactoside (Hyperin)* | Flavonols | 13673667 | 14596667 | 14657667 | 12011333 | 11408667 | 10108833 |
| 6-Hydroxykaempferol-7-O-glucoside | Flavonols | 1987433 | 2099767 | 2024367 | 2043033 | 2033967 | 1807867 |
| Quercetin-3-O-glucoside (Isoquercitrin)* | Flavonols | 13156000 | 16645667 | 14610333 | 12356000 | 11680667 | 10395267 |
| Kaempferide-3-O-glucuronide | Flavonols | 827330 | 461960 | 396976.7 | 89999 | 68898.33 | 88173.33 |
| Quercetin-5-O-glucuronide | Flavonols | 23329533 | 19546067 | 16104767 | 13508667 | 12247667 | 9525567 |
| Tricin-4'-methylether-3'-O-glucoside | Flavonols | 690686.7 | 1380367 | 1510043 | 966100 | 857590 | 724376.7 |
| Isorhamnetin-3-O-Glucoside* | Flavonols | 646373.3 | 1691083 | 2930100 | 127103.3 | 170523.3 | 215693.3 |
| Rhamnetin-3-O-Glucoside* | Flavonols | 725080 | 1200303 | 1325310 | 922516.7 | 927796.7 | 745940 |
| Gossypetin-3-O-glucoside | Flavonols | 16375667 | 8429200 | 4998933 | 1025903 | 530830 | 343893.3 |
| Myricetin-3-O-glucoside | Flavonols | 15835000 | 8124833 | 4596833 | 1066030 | 535976.7 | 341176.7 |
| Kaempferol-3-O-(2''-acetyl)glucoside | Flavonols | 3764600 | 1529600 | 834850 | 511556.7 | 306953.3 | 180706.7 |
| Quercetin-3-O-(6''-acetyl)glucoside | Flavonols | 24209000 | 18362333 | 13208000 | 1117240 | 703246.7 | 357450 |
| Quercetin-3-O-(6''-acetyl)galactoside | Flavonols | 2161533 | 3974767 | 2712667 | 3536200 | 3107167 | 2300567 |
| Quercetin-3-O-(2''-acetyl)glucuronide | Flavonols | 14332433 | 13815667 | 11067800 | 2070467 | 2222933 | 2195733 |
| Isorhamnetin-3-O-(6''-acetylglucoside) | Flavonols | 12886400 | 17018000 | 11640767 | 1596733 | 1790733 | 1774967 |
| Kaempferol-3-O-(6''-malonyl)galactoside | Flavonols | 583570 | 837356.7 | 652520 | 2486667 | 2295167 | 2496533 |
| Kaempferol-3-O-(6''-malonyl)glucoside | Flavonols | 18035000 | 24292000 | 19942333 | 57396667 | 56891333 | 58309333 |
| Quercetin-7-O-(6''-malonyl)glucoside | Flavonols | 2022997 | 1738603 | 1111790 | 3914433 | 3467500 | 2929767 |
| Quercetin-3-O-(6''-malonyl)galactoside | Flavonols | 6938600 | 7355833 | 6432167 | 9844767 | 8092433 | 7288267 |
| Gossypetin-3-O-(6''-malonyl)glucoside | Flavonols | 45957.67 | 105797.7 | 63649 | 162760 | 150163.3 | 148120 |
| Myricetin-3-O-(6''-malony)glucoside | Flavonols | 204606.7 | 108768.7 | 78358 | 9 | 9 | 9 |
| Quercetin-3-O-xylosyl(1→2)arabinoside | Flavonols | 568576.7 | 313766.7 | 172409.3 | 22592.67 | 25831.67 | 16410.23 |
| Kaempferol-3,7-O-dirhamnoside (Kaempferitrin) | Flavonols | 913693.3 | 629316.7 | 459876.7 | 1577600 | 1170267 | 947820 |
| Kaempferol-3-O-(6''-p-Coumaroyl)glucoside (Tiliroside) | Flavonols | 190657 | 771129 | 928885.3 | 9 | 9 | 9 |
| Kaempferol-3-O-neohesperidoside | Flavonols | 694570 | 554376.7 | 466130 | 21262667 | 18817000 | 15970667 |
| Kaempferol-3-O-robinobioside(Biorobin) | Flavonols | 1839533 | 1184133 | 1033990 | 526123.3 | 343160 | 301766.7 |
| Quercetin-3-O-rutinoside (Rutin) | Flavonols | 301050 | 365336.7 | 584653.3 | 1364833 | 1259333 | 1086147 |
| Quercetin-3-O-neohesperidoside | Flavonols | 842446.7 | 486273.3 | 373793.3 | 9 | 9 | 9 |
| Quercetin-3-O-robinobioside | Flavonols | 409383.3 | 361826.7 | 243600 | 9 | 9 | 9 |
| Sexangularetin-3-O-glucoside-7-O-rhamnoside | Flavonols | 4201867 | 3101133 | 2916000 | 10599667 | 11264000 | 11344667 |
| Isorhamnetin-3-O-neohesperidoside | Flavonols | 310216.7 | 300603.3 | 247090 | 9 | 9 | 9 |
| 6-Hydroxykaempferol-7,6-O-Diglucoside | Flavonols | 7184767 | 9181400 | 12099267 | 16497333 | 13462333 | 12449667 |
| 6-Hydroxykaempferol-3,6-O-Diglucoside | Flavonols | 15001667 | 11738700 | 10067533 | 6472433 | 6649033 | 6007900 |
| Quercetin-3-O-(2''-O-galactosyl)glucoside | Flavonols | 266590 | 502563.3 | 439996.7 | 329063.3 | 386343.3 | 243063.3 |
| Isorhamnetin-3,7-O-diglucoside | Flavonols | 1643847 | 2660133 | 4506400 | 353436.7 | 606376.7 | 931266.7 |
| Limocitrin-3-O-(3-hydroxy-3-methylglutarate)glucoside | Flavonols | 2116730 | 4605830 | 5950153 | 9 | 9 | 9 |
| Gossypetin-3-O-glucuronide-8-O-glucoside | Flavonols | 730640 | 1664307 | 3154650 | 1004630 | 1600433 | 2323900 |
| Quercetin-7-O-(6''-malonyl)glucosyl-5-O-glucoside | Flavonols | 645093.3 | 650780 | 546406.7 | 4723933 | 4301100 | 3539400 |
| Quercetin-3-O-(6''-malonyl)glucosyl-5-O-glucoside | Flavonols | 609566.7 | 1623983 | 1481083 | 327426.7 | 387343.3 | 387350 |
| Natsudaidain-3-O-(3-hydroxy-3-methylglutarate)glucoside | Flavonols | 1725.363 | 63489.84 | 117071.3 | 9 | 9 | 9 |
| Isorhamnetin-3-O-(6''-malonylglucoside)-7-O-glucoside | Flavonols | 9657500 | 15654000 | 13048333 | 6337767 | 7270567 | 6704500 |
| Quercetin-3-O-(2''-O-arabinosyl)rutinoside | Flavonols | 71531.67 | 39053 | 40154.33 | 9 | 9 | 9 |
| Quercetin-3-O-(2''-O-Rhamnosyl)rutinoside | Flavonols | 1681900 | 1100900 | 939406.7 | 9 | 9 | 913.2667 |
| 6-Hydroxykaempferol-3-O-rutin-6-O-glucoside | Flavonols | 22185 | 9 | 9 | 29397 | 24652.33 | 23605.67 |
| 6-Hydroxykaempferol-3,7,6-O-triglycoside | Flavonols | 9 | 22406.67 | 41344 | 90350 | 82525.33 | 62532 |
| Apigenin-8-C-Glucoside (Vitexin)* | Flavonoid carbonoside | 22109.67 | 26988.33 | 57120.33 | 1183350 | 714470 | 491886.7 |
| Apigenin-6-C-glucoside (Isovitexin)* | Flavonoid carbonoside | 25693.67 | 26479.33 | 53561.67 | 1176467 | 778240 | 461696.7 |
| Isohemiphloin | Flavonoid carbonoside | 210236.7 | 213230 | 197080 | 228500 | 155423.3 | 137094 |
| Luteolin-6-C-glucoside (Isoorientin)* | Flavonoid carbonoside | 3516.033 | 6249.533 | 7557.267 | 66258 | 49028.33 | 35968 |
| Luteolin-8-C-glucoside (Orientin)* | Flavonoid carbonoside | 6995.433 | 6548.067 | 10481.17 | 159633.3 | 135536.7 | 96033.33 |
| Isoschaftoside | Flavonoid carbonoside | 39862 | 16729.33 | 6969.6 | 4043.933 | 9 | 9 |
| Apigenin-6-C-(2''-glucuronyl)xyloside | Flavonoid carbonoside | 145601 | 146744 | 95563 | 274663.3 | 227740 | 185583.3 |
| Vitexin-2''-O-glucoside | Flavonoid carbonoside | 176763 | 262437 | 194790 | 234226.7 | 248786.7 | 165520 |
| Hesperetin-6-C-glucoside-7-O-glucoside | Flavonoid carbonoside | 1534600 | 1846167 | 1694633 | 5427533 | 5212167 | 4642767 |
| Hesperetin-8-C-glucoside-3'-O-glucoside | Flavonoid carbonoside | 2839033 | 3745367 | 4989200 | 6753800 | 5927000 | 4933967 |
| Cirsimaritin-8-C-[glucosyl-(1-2)]-glucoside | Flavonoid carbonoside | 9 | 9 | 9 | 263196.7 | 193293.3 | 124486.7 |
| 5,7,3',4'-Tetrahydroxy-6-methoxyflavone-8-C-[glucosyl-(1-2)]-glucoside | Flavonoid carbonoside | 237243.3 | 298480 | 462270 | 62125.33 | 122754.7 | 150973.3 |
| Isovitexin-7-O-(6''-feruloyl)glucoside | Flavonoid carbonoside | 98135 | 234446.7 | 233503.3 | 9 | 9 | 9 |
| Epicatechin* | Flavanols | 4807300 | 2452633 | 1518233 | 5794233 | 4439000 | 2949200 |
| Catechin* | Flavanols | 15758000 | 2685867 | 913960 | 16388000 | 6703067 | 2317100 |
| 3'-O-Methyl-(-)-epicatechin | Flavanols | 682856.7 | 663286.7 | 484150 | 2124200 | 2424233 | 2501933 |
| Epigallocatechin* | Flavanols | 2707967 | 727810 | 230670 | 1075987 | 73179 | 85954.67 |
| Gallocatechin* | Flavanols | 15697333 | 3611533 | 719780 | 5525333 | 488173.3 | 338326.7 |
| Catechin-(7,8-bc)-4β-(3,4-dihydroxyphenyl)-dihydro-2-(3H)-one | Flavanols | 4640500 | 5073733 | 5117700 | 988266.7 | 1094683 | 1056967 |
| Catechin-(7,8-bc)-4α-(3,4-dihydroxyphenyl)-dihydro-2-(3H)-one | Flavanols | 18026733 | 10167767 | 7126067 | 3301633 | 3153300 | 1894700 |
| Epicatechin glucoside | Flavanols | 229046.7 | 162593 | 153056.7 | 1079340 | 1007080 | 763220 |
| Epicatechin-epiafzelechin | Flavanols | 110242.7 | 95918.33 | 69290.33 | 205600 | 187886.7 | 141460 |
| Gallocatechin-(4α→8)-catechin | Flavanols | 50697.67 | 31154.33 | 27714.67 | 14578.2 | 9538.1 | 8687.767 |
| Catechin-catechin-catechin | Flavanols | 3271567 | 2723867 | 1818367 | 3694433 | 3327000 | 2441400 |
| Formononetin (7-Hydroxy-4'-methoxyisoflavone) | Isoflavones | 1117.793 | 1054.063 | 1034.57 | 1420.133 | 1008.777 | 893.2633 |
| Prunetin (5,4'-Dihydroxy-7-methoxyisoflavone) | Isoflavones | 20480.33 | 39228.67 | 68839.33 | 12783 | 18187.33 | 20716 |
| Genistein-8-C-glucoside | Isoflavones | 59083.67 | 103024 | 150096.7 | 3224700 | 2263733 | 1408967 |
| Biochanin A-7-O-glucoside (Sissotrin) | Isoflavones | 28810.33 | 10395.93 | 10848.07 | 3768.967 | 2795.167 | 9 |
| Iridin | Isoflavones | 127622.3 | 285100 | 330553.3 | 83191 | 122063.3 | 142986.7 |
| Genistein-7-O-galactoside-rhamnose | Isoflavones | 879413.3 | 584573.3 | 484690 | 1569667 | 1095710 | 927223.3 |
| 2'-Hydoxy,5-methoxyGenistein-O-rhamnosyl-glucoside | Isoflavones | 3709333 | 3037033 | 2491133 | 9972767 | 9072500 | 10513800 |
| Procyanidin B4 | Proanthocyanidins | 1931867 | 1164577 | 1154953 | 2912133 | 2476700 | 1569900 |
| Procyanidin B2 | Proanthocyanidins | 30994000 | 24721333 | 19000000 | 33874333 | 29247667 | 24495333 |
| Procyanidin B3 | Proanthocyanidins | 7461433 | 3337367 | 1700667 | 4016867 | 3613100 | 2464067 |
| Procyanidin B1 | Proanthocyanidins | 13629667 | 10674733 | 6455667 | 16299333 | 11900333 | 9285967 |
| Procyanidin C1 | Proanthocyanidins | 2507367 | 2084233 | 1533733 | 2777800 | 2470833 | 1906100 |
| Gallocatechin-catechin-catechin | Proanthocyanidins | 380406.7 | 164320 | 64773 | 39730.33 | 17456.13 | 9 |
| Gallocatechin-gallocatechin-catechin | Proanthocyanidins | 133148.7 | 56930 | 19621.67 | 183083.3 | 92299.67 | 44553 |
