# Supplementary material for: Integrated LC-MS/MS and Transcriptome Sequencing Analysis Reveals the Mechanism of Color Formation During Prickly Ash Fruit Ripening
Source: Front Nutr. 2022 Mar 16;9:847823. doi: 10.3389/fnut.2022.847823 (PMC8967253; doi:10.3389/fnut.2022.847823)
Supplement: Supplementary file 2 [file Table_2.DOCX]

**Table S2.** The content of 10 flavonoids in different growth periods of red and green prickly ash fruit.

| Class | R1 | R2 | R3 | G1 | G2 | G3 |
| --- | --- | --- | --- | --- | --- | --- |
| Chalcones | 3350623.90 | 1157021.50 | 811871.67 | 417037.47 | 192622.87 | 132295.83 |
| Dihydroflavone | 18014192.57 | 10909052.50 | 8802153.27 | 5916904.47 | 4288487.07 | 3011752.47 |
| Dihydroflavonol | 2542338.90 | 2288423.63 | 2075703.97 | 2470221.33 | 2521797.67 | 2113278.67 |
| Anthocyanins | 12218533.33 | 79320830.00 | 241846336.33 | 4176297.00 | 2175277.00 | 1607937.00 |
| Flavonoid | 195495933.23 | 247696727.77 | 266955981.50 | 115427516.57 | 124556417.59 | 129089957.80 |
| Flavonols | 272752279.63 | 260238723.54 | 222807311.03 | 209010353.90 | 197104316.23 | 182856847.53 |
| Flavonoid carbonoside | 5339798.13 | 6829874.60 | 8002738.70 | 15833806.27 | 13764457.67 | 11425993.33 |
| Flavanols | 65982243.67 | 28396162.33 | 18178988.33 | 40191604.87 | 22907140.43 | 14498949.10 |
| Isoflavones | 4825861.13 | 4060409.33 | 3537195.30 | 14868296.43 | 12575997.94 | 13014594.93 |
| Proanthocyanidins | 57037888.67 | 42203493.33 | 29929414.67 | 60103280.33 | 49818389.13 | 39765928.67 |
