# Supplementary material for: Integrated LC-MS/MS and Transcriptome Sequencing Analysis Reveals the Mechanism of Color Formation During Prickly Ash Fruit Ripening
Source: Front Nutr. 2022 Mar 16;9:847823. doi: 10.3389/fnut.2022.847823 (PMC8967253; doi:10.3389/fnut.2022.847823)
Supplement: Supplementary file 3 [file Table_3.DOCX]

**Table S3.** Genes of terpenoid synthesis pathway.

| **Gene Symbol** | **Description** | **ID** |
| --- | --- | --- |
| *PAL1* | Phenylalanine ammonia-lyase | EVM0076872 |
| *PAL2* |  | EVM0012211 |
| *C4H1* | Cinnamic acid hydroxylase | EVM0005415 |
| *C4H2* |  | EVM0091380 |
| *4CL1* | 4-coumarate-CoA ligase | EVM0059348 |
| *4CL2* |  | EVM0083091 |
| *CHS1* | Chalcone synthase | EVM0068077 |
| *CHS2* |  | EVM0095657 |
| *CHI1* | Chalcone isomerase | EVM0078924 |
| *CHI2* |  | EVM0070967 |
| *F3H1* | Flavanone 3-hydroxylase | EVM0075724 |
| *F3H2* |  | EVM0078812 |
| *F3'H1* | Flavonoid 3′-monooxygenase | EVM0016522 |
| *F3'H2* |  | EVM0012078 |
| *F3'5'H1* | Flavonoid 3′,5′-hydroxylase | EVM0089749 |
| *F3'5'H2* |  | EVM0070244 |
| *ANS1* | Anthocyanidin synthase | EVM0001689 |
| *ANS2* |  | EVM0085766 |
| *DFR1* | Bifunctional dihydroflavonol 4-reductase | EVM0083699 |
| *DFR2* |  | EVM0032407 |
| *UFGT1* | UDP-glucose flavonoid 3-O-glucosyl transferase | EVM0073358 |
| *UFGT2* |  | EVM0053212 |
